# Subjective Perceptions of ‘Meaning of Work’ of Generation MZ Employees of South Korean NGOs

**DOI:** 10.3390/bs13060461

**Published:** 2023-06-02

**Authors:** Sangsuk Moon, Yucheon Kim

**Affiliations:** Department of Counselling and Coaching, Dongguk University, Seoul 04620, Republic of Korea; sangsukmoon@dgu.ac.kr

**Keywords:** meaning of work, NGO, generation MZ, Q methodology, subjective perceptions

## Abstract

This study used the Q methodology to analyse the perceptions of the meaning of work among Generation MZ employees working for South Korean nongovernmental organisations (NGOs). Forty Q samples about the meaning of work were extracted through a literature review and in-depth interviews, and 24 Generation MZ employees working for NGOs were selected as P samples to carry out Q sorting. The results were analysed using the KenQ program, and the perceptions of the meaning of work among Generation MZ employees working for NGOs were classified into four types. For Type 1, work was perceived as a means of self-realization corresponding to their values and an opportunity for new challenges. Type 2 employees expect people to recognise them as people of value through their work and pursue the satisfaction of contributing to people and society through their employment. Type 3 employees expected work to be a happy and interesting experience that coincided with their values beyond simple money-making. Finally, Type 4 considered work and personal life more separate and prioritised solidarity with colleagues.

## 1. Introduction

Despite the public nature of NGOs, what is the meaning of work for generation MZ in their workplaces? It is generally assumed that it would be very different from the meaning of work in the workplaces of previous generations. To this end, the researchers aim to examine the meaning of work for generation MZ by first looking at the meaning of NGOs and the meaning of generation MZ. Although there are various definitions of nongovernmental organisations (NGOs), they generally refer to voluntary organisations that aim to achieve a public purpose and carry out activities to pursue public interests in the non-profit sector rather than the government or the market [1,2,3]. The roles of NGOs that work for the public interest of our society are gradually expanding so that they not only check the power of the government and represent the rights and interests of the socially underprivileged but also perform the functions of policy execution, conflict resolution, and social education and conduct diverse activities to realise public interest values [2]. With the recent expansion of civic consciousness, changes in the role of the government, and expansion of governance, NGOs are steadily growing and developing in South Korea as well. According to the statistics of the Ministry of Public Administration and Security for 2021, there will be more than 15,000 registered non-profit private organisations that operate with more than 100 employees. NGOs have limitations on the use of human resources due to financial constraints that depend on donations. In particular, given the low wages and high turnover of NGO employees, the degree of commitment and resignation rate of members are key management indicators for the stability of organisational management and sustainable growth. In addition, a crucial research task for NGO business management is to determine how to continuously motivate individuals and combine the goals of individuals and the organisation to strengthen the feeling of solidarity.

Despite the publicity of NGOs, the era of forcing social commitment onto Generation MZ, which refers to “Millennials” and “Generation Z”, has ended. There is no clear standard set for Generation MZ. Millennials first appeared in Strauss et al.’s [4] book Generations: The History of America’s Future. The authors first used the term “millennial generation” and defined it as people born between 1982 and 2004. Generation Z is the generation after the Millennial generation and includes those born from the mid-1990s to the mid-2000s. They are also called “digital natives” because they grew up in a digital environment and have owned mobile phones since they were young [5]. Generation Z, or Gen Z, or iGen, or post-millennials (born between 1995 and 2012) is the latest generation to join the workforce in 2017 [6]. This study uses the term “Generation MZ” to refer to those who were born between 1981 and 2009, following the criteria of [7].

Unlike previous generations, that is, Generation X and the Baby Boom generation, who devoted themselves to the interests and future of the organisation, Generation MZ, represented by “work-life balance” and “quiet resignation”, actively moves in search of organisations that are harmonised with their values and enable self-realisation [8]. Generation MZ, who are familiar with digital media from an early age, are comfortable with the free communication style that digital media enables. Therefore, they want to communicate their personal values more freely and actively in organisations [9]. In addition, Generation MZ, which experienced low economic growth, is a generation that considers their own subjective perceptions, such as the meaning or value of work and work-life balance, more important than objective indicators, such as high salaries and positions [10]. They do not live according to standards determined by others, and they pursue self-satisfying happiness and success [11]. They place the most value on themselves [12], think of themselves before work or the workplace, and prioritise their lives and happiness over everything else [13]. Generation MZ members regard their career as important and have a strong desire to develop their expertise through work [14], but they do not hesitate to leave the organisation they belong to if they believe that the work they do is not conducive to their career [15].

Despite the intensifying difficulties youths face in job seeking, Generation MZ’s resignation rate continues to increase. According to a 2021 survey with 500 companies conducted by the employment platform “Saramin”, the “rate of resignation within one year of new employees” was 28%, representing a more than 10% increase compared to that of 2019 (17.9%). Moreover, it was shown that the rate of resignation in Generation MZ was particularly high and that 5 out of 10 could not last one year and resigned earlier. The results of a 2021 survey conducted with 343 male and female office workers in their 20s and 30s by another employment platform, “Job Korea”, were similar. It was found that more than 3 out of 10 Generation MZ employees resigned less than a year after joining the company; at least half resigned within two years, and at least 90% resigned within five years. The situation in NGOs is not much different, and in the case of Child Rights Agency S, the cumulative resignation rate from 2020 to 2021 was 19.4%. The resignation rate is showing an increasing trend every year, and most of those who resign are staff members, assistant managers, and managers in their 20s or 30s, members of Generation MZ. The resignation of an organisation member not only interrupts the workflow and delays the organisation’s scheduled decision-making but also negatively affects colleagues [16]. Furthermore, the loss of competent employees leads to increased costs and reduced performance at the organisational level, which can eventually hinder the productivity and profitability of the organisation [17].

Due to the nature of South Korean NGOs, where wages and employee benefits are low, the stability of human resources is even more important. To reduce the resignation rate of Generation MZ members working for NGOs and achieve stable growth and development, it is necessary to focus on members’ perceptions of the “meaning of work”, or what work means to an individual and what role it plays in his or her life [18]. The meaning of work positively affects not only attitudes and behaviours toward work but also organisational effectiveness, that is, organisational members’ commitment to work [19,20], organisational commitment [21], job satisfaction [22], and innovative behaviour [23]. Examining the “meaning of work” will provide an in-depth understanding of the characteristics of employees and an organisational management plan suitable for those characteristics.

Scholars’ definitions of the meaning of work are diverse, but they can be largely divided into two dimensions, the first of which is the cognitive dimension. Chalofsky [24] defined the meaning of work as not only the reward for the work carried out by the person but also the alignment of the purpose, values, and relationships pursued in life. Jang [25] defined the meaning of work as the cognitive evaluation subjectively given by the person to the work being done by the individual. Finally, Rosso et al. [18] defined the meaning of work as what work means in individuals’ lives and what roles it occupies in their lives. The second dimension of the definition of the meaning of work is the cognitive-behavioral dimension, which extends the meaning of work to experiences and attitudes beyond the level of individual perception. Steger et al. [26] defined the meaning of work as regarding one’s work as important and as a subjective experience of finding oneself and growing through work while the work is positively influencing others or society. Tak et al. [27,28] defined the meaning of work as the totality of the beliefs, values, motives, importance, and purpose that an individual has about work, that is, a comprehensive attitude of cognition, emotion, and behaviour toward work. Finally, Kim et al. [29] saw meaningful work as work that not only brings about economic rewards but also makes individuals’ lives meaningful.

The definitions of the meaning of work are shown in Table 1 below. 

Work is a basic activity in human life [30] and not only brings about economic rewards but also makes individuals’ lives meaningful [29]. People want the work in which they invest so much of their time and energy to be more meaningful than just a means to earn money; through work, humans construct their identity and social meanings [31]. As society has advanced, work has become linked to self-identity and established as a basis for self-esteem [32]. Thus, work is now not only a means of maintaining an individual’s life but also a reference point that creates a concrete reality that makes an individual’s life valuable.

Regarding the effect of the meaning of work, Wrzesniewski et al. [33] stated that people who think that their work is meaningful have low job stress and have positive ways of coping with job stress so that they positively cope with and find ways to solve problems. According to the conservation of resources theory, stress occurs in situations where individuals may lose resources they value, actually lose resources, or fail to obtain as much as they want. And people are more sensitive to losing than gaining resources [34]. Moreover, a study conducted by the Samsung Economics Research Institute, which investigated factors that affect office workers’ happiness, found that the meaning of work was the most influential variable in increasing happiness [35].

As people perceive the meaning of their work as greater, they feel more pleasure in their work, their interest in the job increases [36], and job-related burnout is reduced [37]. Eventually, the meaning of work motivates people and affects job satisfaction and job performance, thereby becoming the driving force and source of happiness that enables individuals to actively demonstrate their capabilities.

This study aims to understand the perceptions of the meaning of work among Generation MZ employees working for NGOs. The theoretical basis of Q methodology lies in Professor William Stephenson’s Concourse Theory (1978), The Play Theory of Mass Communication (1967), and the Quantum Theory of Subjectivity (1988), as well as self-psychology, inter-behaviouralism, hermeneutics, and others. Within the individual’s subjective behaviour, all concepts, associations, ideas, etc. are perceived and expressed, and there exists a discourse as a sum of shared knowledge. The self-referential subjectivity and factor structure of the Q methodology provide an opportunity to generate hypotheses that are inherent in the concourse. Through this, one can discover the relativity of cultures and values [38].

Created by Stephenson in 1935, the Q methodology, a study method aimed at grasping the cognitive structure of the mind by directly asking individuals about their thoughts on a certain topic, is useful to objectively view individuals’ different attitudes and experiences and enables in-depth measurement of human subjectivity [39]. Unlike quantitative study methods that deal with external phenomena, the Q methodology provides a foundation for considering the subjective viewpoints, views, opinions, beliefs, and attitudes perceived by each individual about a certain object or situational context [40].

By exploring the subjective perceptions of the “meaning of work” of Generation MZ employees of NGOs, categorising them into types, and investigating the characteristics of each type, this study will improve our understanding of the methods suitable for motivating them. It is hoped that the findings will be utilised as meaningful basic data in reducing the employee resignation rate of NGOs, whose social roles are continuously increasing, and increasing employee satisfaction.

To this end, the research questions are as follows: Research question 1: What are the types of Generation MZ employees of NGOs categorised by their perceptions of the meaning of work?

Research question 2: What are the characteristics of each type of Generation MZ employee of NGOs categorised by their perceptions of the meaning of work? 

## 2. Materials and Methods

This study utilised the Q methodology as follows: First, Q populations were constructed by collecting data through previous research, data analysis, and individual in-depth interviews. Q samples were selected through a review by four experts, and 24 members of Generation MZ working for NGOs were selected as P samples. Q sorting was conducted with the forced distribution method by P samples with an 11-point scale, and the sorted data were analysed using KenQ v1.0.8. The overall study process is shown in Table 2. 

Q populations are collections of statements for the Q study. Constructing Q populations involves collecting all opinion statements [38]. Q populations are self-referential and should be statements about personal opinions rather than facts, into which the respondents can project themselves [39]. As methods to collect Q populations, literature reviews, questionnaires, and in-depth interviews are mainly used. In this study, a total of 95 statements were extracted through the literature review and review of newspaper articles and reports involving searches for keywords related to the “meaning of work” or “meaning of job” of NGO employees. Second, a total of 113 Q populations were constructed by additionally collecting 37 statements through Q questionnaire surveys and Q in-depth interviews.

Q samples refer to the statements extracted from Q populations. Q statements are sentences that express people’s opinions, feelings, thoughts, and actions on the research topic [41]. The Q samples in this study were subjective statements on the perceptions of the meaning of work by Generation MZ employees of NGOs. To prevent overlapping Q sample selection, the Q statements were categorised into six types: economics, self-realisation, happiness, social, quality of life, and others. Tak et al.’s [28] study on the Work Meaning Inventory was referred to in order to remove overlaps, and 40 statements were finally selected. The final Q samples are shown in Table 3.

P samples are those who sort the statements (Q samples) extracted from the P populations. The purpose of the Q methodology is not a generalisation. The R method needs a lot of samples, but Q-sorting only needs a few people. In Q, the number of people is enough if you can create and compare factors. It is usual to have between 10 and 100 people at most [42]. Therefore, the P samples in this study consisted of 24 Generation MZ members working for NGOs. Most NGO workers in Korea are women, and only about 20% are men. As for the male-to-female ratio of the P samples, there was a significantly higher proportion of females (20) than males (4). The distribution by age group consisted of 5 samples in their 20s, 13 samples in their 30s, and 6 samples in their 40s as shown in Table 4. Prior to data collection, the purpose and process of this study were fully explained to the P samples, and Q sorting was carried out after obtaining consent.

**Figure 1:**
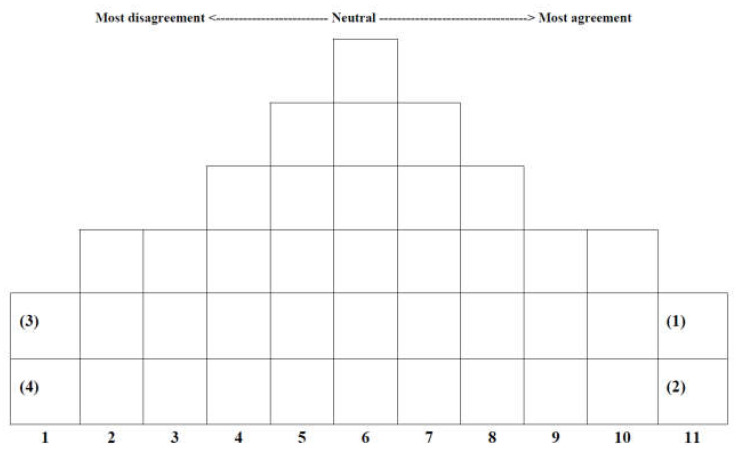
Q distribution chart.

Q sorting is a process through which P samples place the Q samples on the given allocation table in the order in which they agree according to the individuals’ viewpoint. Therefore, there is no researcher’s evaluation standard for Q sorting, and the purpose of Q sorting is to examine how the statements of the Q samples are distributed and stratified by each individual P sample [43]. In this study, after the Generation MZ members selected as P samples were requested to read the 40 statements, they were asked to first sort the statements into three levels of agree, neutral, and disagree according to their degree of agreement. They were then asked to sort the statements on the Q sample distribution chart in Figure 1 according to their degree of agreement with each statement. Thereafter, additional questions were asked about the items of the statements placed at both extremes, and the answers were used to analyse the perceptions of the “meaning of work” of Generation MZ employees working for NGOs.

Q sorting is normalised by a method of forcing the distribution of Q statements, and the mean is 0 and the standard deviation is also the same. The distribution of Q sorting varies depending on the number of statements, and it is common to use a 9-point scale if the number of statements is less than 40 and an 11-point scale if it is more than 40 [38]. The collected data were scored on an 11-point scale by checking the statement numbers recorded on the Q sample distribution chart and were coded thereafter. To analyse the data, the standard scores of individual items were obtained, and principal component factor analyses were conducted using the PC KenQ program. Based on an eigenvalue of 1.0 or higher, the numbers of factors were input diversely, and out of the results calculated as such, four factors that best showed the differences by type were selected. Subsequently, standard scores (Z-scores) were used to select appropriate statements by type, and the reasons for selecting the statements with the most agreement and disagreement centred on subjects with high factor weights by type were used to interpret the characteristics by type.

## 3. Results

### 3.1. Result Analysis

The Q factors were analysed using the PC KenQ program, and based on the results, four factors among the factors with eigenvalues, which are factor eigenvalues, of 1.0 or higher were judged to be the most suitable for categorization. The explanatory power of the four factors was 64% in total, as shown in Table 5 below, and the eigenvalues for Type 1, Type 2, Type 3, and Type 4 were 9.8081, 2.5302, 1.5321 and 1.389, respectively. As for the variances explained by individual types, Type 1, Type 2, Type 3, and Type 4 accounted for 41%, 11%, 6%, and 6%, respectively.

The correlation coefficients that showed the similarity between individuals were 0.538 for Type 1 and Type 2, 0.5348 for Type 1 and Type 3, 0.4911 for Type 1 and Type 4, 0.5906 for Type 2 and Type 3, 0.3997 for Type 2 and Type 4, and 0.4603 for Type 3 and Type 4, as shown in Table 6 below.

A factor weight is calculated for each individual through factor extraction and rotation. It can be seen that an individual with a higher factor weight is more representative of the factor or type [38]. Three (P14, P8, and P17) of the 24 P samples in total showed significant factor weights for at least two factors. Since this was also interpreted as the absence of a decisive factor showing the typicality of the P samples, the P samples were excluded from this study [44]. The subjects by type and type weights are shown in Table 7 below. In this study, a total of 21 P samples were sorted into a total of four types: 4 into Type 1, 7 into Type 2, 3 into Type 3, and 7 into Type 4. Moreover, among factor weights of P samples by type, P15 showed the highest factor weight at 10 for Type 1, P20 at 12.9776 for Type 2, P10 at 17.5581 for Type 3, and P13 at 20.3488 for Type 4.

### 3.2. Perception Type Characteristics

#### 3.2.1. Type 1: ‘Work Is My Opportunity to Grow’

Type 1 was named “Work is my opportunity to grow”. Type 1 samples recognised work as a means of self-realisation that coincides with their values and an opportunity for new challenges, although they did not consider their value at work to be evaluated as a result of their work. When Type 1’s standard scores by item were examined, it could be seen that Type 1 showed the strongest agreement with Q26. “My value is not evaluated (defined) by the outcome of my work (Z = 1.759)”, followed by “Q9. I am happy only when my work fits my values (Z = 1.655) and Q16. Work is an opportunity to try new things (Z = 1.617), in order of precedence. On the other hand, Type 1 strongly disagreed with Q27. If I could continue to receive unemployment benefits, I would not bother to work (Z = −1.853) and “Q7”. If I were born with a silver spoon in my mouth, I would not bother to work. (Z = −1.742)’. In addition, Type 1 showed points of differentiation from other types, such as Q26. “My value is not evaluated (defined) by the outcome of my work (Z = 1.759)”, Q16. Work is an opportunity to try new things (Z = 1.617), Q37. I want to continue working beyond retirement age (Z = 1.003) and Q30. One’s job determines one’s social status (Z = −1.43). On the other hand, P15 (10), who had the highest factor weight as a typical example of Type 1, saw “work as an opportunity to improve his skill level, as he can gain experience in the areas in which he is lacking” and said, “I want to become an expert in my field and work for a long time regardless of money, like designers who work long after they have hit retirement age”. P24 (9.973), who had the second highest factor weight, said, “I think self-realisation through work is possible”. I think I can have new relationships, thoughts, and views that I have not experienced before, and I can get the opportunity to nourish myself by experiencing new projects in the course of my work. The details of the scores by Type 1 item are shown in Table 8 below.

#### 3.2.2. Type 2: ‘Work Enables Me to Realise My Value’

Type 2 was named “Work enables me to realise my value”. Type 2 samples are expected to recognise themselves as people of value through work and perceive work as an opportunity to feel the satisfaction and sense of achievement of contributing to people and society. To consider themselves valuable and feel a sense of satisfaction, achievement, and happiness, they regarded doing work that fits their abilities as more important than the other types. Type 2 samples showed the strongest agreement with Q9. “I am happy only when my work fits my values (Z = 1.814)”, followed by “Q10”, “Work enables me to feel that I am a person of value (Z = 1.777)”, and “Q17”. Work gives me the feeling of satisfaction that I am contributing to society and people (Z = 1.679) and “Q13. Work is an opportunity to feel a sense of achievement” (Z = 1.41). On the other hand, Type 2 disagreed the most strongly with Q7. If I were born with a silver spoon in my mouth, I would not bother to work (Z = −2.016) and show strong tendencies to disagree with Q27. If I could continue to receive unemployment benefits, I would not bother to work (Z = −1.566) (Q.23). Work cannot make me remain the way I am (Z = −1.549) and “Q38”. It’s important that I do work that fits my ability (Z = −1.26). P20 (12.978), who had the highest factor weight as a typical example of Type 2, said, “I can be forced to do work that does not fit my values for a living, but I don’t think it satisfies my needs other than my financial needs”. I think I should be able to find meaning, even if it is small, to be able to find a driving force to keep doing the work, and I think work should be meaningful, not only from a personal point of view but also from a social point of view. The details of the scores by Type 2 item are shown in Table 9 below.

#### 3.2.3. Type 3: ‘Work Is an Interesting Experience’

Type 3 was named “Work is an interesting experience”. Type 3 samples considered work, which takes up at least one-third of their lives, something that determined the kind of life they have. Since so much time is spent on work, they expected work to be a happy and interesting experience that coincided with their values beyond simple money-making. Type 3 samples wanted to be interested in their field of work, meetings with various people, and the processes required to achieve results. They showed the strongest agreement with Q9. I am happy only when my work fits my values (Z = 2.028), followed by “Q29”. It is important to work with people who share the same values and beliefs (Z = 1.846), Q24. Work should be interesting (Z = 1.543), Q31. I am happiest when I work with a sense of duty (Z = 1.541), Q19. Work should be meaningful (Z = 1.485) and “Q28. Work is a place to meet, communicate, and exchange with various people” (Z = 1.284). The points of differentiation from the other types were shown to be as follows: “Q38”. It’s important that I do work that fits my ability (Z = 1.225) and enhances my self-esteem (Z = 0.974). On the other hand, they disagreed the most strongly with Q23. Work cannot make me remain the way I am (Z = −1.907) and showed a strong tendency to disagree with Q11. It is hard for me to feel that I am growing through my work (Z = −1.543). P10 (17.558), who had the highest factor weight as a typical example of Type 3, said, “Work enables meetings with various people for purposes beyond simple money-making”. “Leading a social life and belonging to a group consisting of people of different backgrounds, genders, and ages is an interesting experience. If I’m working, it must be interesting for me”. The details of the standard scores by Type 3 item are shown in Table 10 below.

#### 3.2.4. Type 4: ‘Work Is Just a Part of Life’

Type 4 was named “Work is just a part of life”. Type 4 samples thought, “Work is just a part of my life, and although it can help me grow personally, the company’s growth is not equated with my growth”. Work is fundamentally stressful, so it is important to work with people who share my values and beliefs. Work efficiency increases and personal quality of life is improved when work and personal life are separated. Type 4 samples showed the strongest agreement with Q34. Work is only a part of life (Z = 2.201), followed by Q9. “I am happy only when my work fits my values (Z = 1.82)”, Q29. It is important to work with people who share your values and beliefs (Z = 1.753) and Q35. To work well, I need the time to be fully invested in myself (Z = 1.37). On the other hand, they disagreed the most strongly with Q27. If I could keep receiving unemployment benefits, I would not bother trying to work (Z = 1.97), and I showed a strong tendency to disagree with Q18. Work is like studying while being paid (Z = 1.658) and “Q7”. If I were born with a silver spoon in my mouth, I would not bother to work (Z = 1.629). P12 (11.998), who had the second highest factor weight as a typical example of Type 4, said, “The greater the immersion in work, the greater the obsession and sense of loss”. P12 also said, “Since “work” takes up a large part of life, I think that sharing values with, trusting, and forming a bond with my colleagues makes me have a sense of emotional stability beyond a sense of solidarity or camaraderie”. On the other hand, regarding working beyond retirement age, he said, “Since time passes, physical and mental ageing is natural, and trends change rapidly; working indefinitely is like punishment”. The details of the standard scores by Type 4 item are shown in Table 11 below.

### 3.3. Consensus Items

Consensus items refer to items with which individual types commonly agree. The total number of consensus items of individual types was shown to be five, as shown in Table 12. Consensus items enable finding commonalities among factors so that one can understand the different characteristics of factors rather than interpreting the characteristics by factor. Statements that responded in the positive direction in the consensus items were “Q9”. “I am happy only when my work fits my values” and “Q3”. “Work enables economic independence”, while statements that responded in the negative direction were “Q27”. “If I could keep receiving unemployment benefits, I would not bother trying to work”. Q11. “It is hard for me to feel that I am growing through my work” and “Q7”. “If I were born with a silver spoon in my mouth, I would not bother to work”. 

## 4. Discussion

This study applied the Q methodology [38]—used to analyse people’s personal experiences with and views on a certain topic, analyse correlations, and categorise people’s statements—to sort MZ members working for NGOs into types based on their perceptions of “the meaning of work”. The results of the study showed a total of four types: Type 1: “Work is my opportunity to grow”, Type 2: “Work enables me to realise my value”, Type 3: “Work is an interesting experience”, and Type 4: “Work is just a part of life”. Accordingly, in addition to the characteristics by type and the differences between characteristics, the commonalities of members of Generation MZ working for NGOs were also examined.

First, the examination of the characteristics of each type revealed that Type 1 samples thought that “work is their opportunity to grow” and that their value was not evaluated by the outcomes of their work. Moreover, although they did not think that “they are their work”, they perceived work as an opportunity to carry out self-realisation that fits their values and try new things. They thought that work improved their abilities, which they had little or no experience in, enabled them to enter into new relationships with people, and expanded their work horizons. For them, work is not stressful but an opportunity for self-realisation and growth, along with economic freedom. Therefore, even if they are free to stop, they hope to continue to seize opportunities for growth and work beyond retirement age. Although they consider work and personal life separate, as a characteristic of Generation MZ, their desire for “growth” is reflected in their work.

Type 2 samples pursued the satisfaction of contributing to people and society through work, as work enabled them to realise their value. If possible, they wanted to do socially meaningful work, and it was very important for them to choose a job that fit their values as such. Therefore, they regarded work as something that made them feel like people of value. The “Work enables me to realise my value” type samples also considered the sense of accomplishment coming from work important and thought that their work reflected them well.

Type 3, named “Work is a Fun Experience”, identified themselves with work to the highest extent. They perceived work and social life as interesting experiences and believed that work made them be themselves and happy through various experiences. Type 3 samples who pursued interest and significance simultaneously thought that their work should coincide with their values since they spend most of their time on work. They also prioritised colleagues who held their values and beliefs because those who shared various experiences were important.

Type 4 samples, named “Work is just a part of life”, faced the unavoidable stress coming from work life squarely and had the strongest desire for a work-life balance. Since work life involves stress, they wanted to do their work well, but they did not want their work to invade their lives too much. They also thought it was important to do work that fits their values and to work with people who have similar values and beliefs. They also believed that in order to work well, they needed to be fully invested in themselves. They did not want to quit their jobs early because of the benefits that work gives them, such as economic independence, pride in their career, and interaction with people, but they did not long to continue working beyond retirement age.

Second, individuals’ viewpoints on work can be divided into three dimensions: jobs, careers, and calling [33,45], and these can be substituted by type. Those who consider work their livelihood (job orientation) are interested in the material benefits that can be obtained through work. Work is not meaningful in itself but as a means of obtaining resources that enable spending time away from work. On the other hand, those who view work as a career (career orientation) make more personal investments in their work and seek status and prestige within their workplace or industry. Those who consider work a calling (calling orientation) perceive work and life as indistinguishable. They think of work as a means of integrating their lives and identities. Moreover, they work for the sense of accomplishment brought about by doing the work rather than for financial gains or career development, and they value the joy of feeling worthwhile. That is, it can be said that approaching work as a job or career is driven by extrinsic motivation while approaching work as a calling is driven by intrinsic motivation. As for the three dimensions, one dimension may be apparent in an individual, or two or three dimensions may appear simultaneously [33].

When individuals are divided into those who see work as a livelihood (job orientation), those who view it as a career (career orientation), and those who see it as a calling (calling orientation), individual types can be examined, as shown in Table 13 below.

Type 1, “Work is my opportunity to grow”, can be said to be included in the job, career, and calling orientations because Type 1 samples thought that their existence could not be evaluated with the outcomes of work. Rather, they pursued economic independence and self-realisation through work and expected growth in the workplace. Type 2, “Work enables me to realise my values”, can be said to be included in the calling orientation because Type 2 samples perceive that work makes them feel the satisfaction of contributing to people and society and feel valuable. Type 3, “Work is a fun experience”, who expected work to be a fun and interesting experience for purposes beyond simple money-making, can be said to be included in the calling orientation because they considered values and beliefs important the most among all types. Type 4, “Work is just a part of life”, separated work from themselves but regarded work as an economic necessity, and since they must work anyway, they wanted to do a job that fit their values and perceived work as a career-building process. Therefore, they can be said to have both job and career orientation dimensions. In the case of Generation MZ employees working for NGOs, although the meaning of work differed by type (e.g., growth, interest, social contribution, and separation of work and personal life), the intrinsic motivation termed “realization of values” was judged to be an important factor in their choice of occupation. The most common statement was “I am happy only when my work fits my values”.

Consequently, it can be said that Type 1 and Type 4 are included in the job orientation because both view work as a means of achieving economic independence. Regarding career orientation, it can be said that Type 1 and Type 4 are included. This is because they recognise work as a process of building the career they want and think they need time to fully invest in themselves to do their job well. With regard to the calling orientation, it can be said that Type 1, Type 2, and Type 3 are included because they view work as a means of growth and realisation of their values and as an important part of their lives. It can be said that a significant number of Generation MZ employees of NGOs view working in NGOs as a calling.

## 5. Conclusions

The significance and implications of this study are as follows: First, this study examined the fact that the perceptions of the meaning of work among Generation MZ employees working for NGOs are diverse. Therefore, it is necessary to continuously communicate with members about the organisation’s beliefs and visions, such as social values pursued by NGOs, personal growth, and respect for individuals. Generation MZ employees of NGOs regard the consensus of the values of individuals and organisations as important, and they want the life and time of individuals to be maintained and the meaning of work pursued by individuals to be respected. Organizations and individuals can grow and develop together when they share the social vision and activities pursued by NGOs while acknowledging the lives, time, and motives of individuals. Organizational vision more strongly motivates members when the members increase organisational commitment and morale and the vision is shared [46].

Second, as shown in the types of perceptions of the meaning of work, Generation MZ employees working for NGOs are more interested in individuals than in organisations. Therefore, NGO organisations need to pay close attention to personal growth management and career development. According to the results of this study, Generation MZ employees of NGOs tend to want to continue “work” as a social activity regardless of their economic conditions. Table 12. Those who feel a sense of meaning in their work show higher intrinsic motives and higher meaning in life [32]. It is necessary to give suitable opportunities to individuals and motivate them appropriately by type so that their self-motivation is not lost or deteriorated in the course of performing work.

Third, despite the rapid growth of NGOs, there are few systematic studies on NGO members’ labour management. Although mainly quantitative studies have been conducted thus far, to view organisational members as active beings with autonomy and help them to form meaning and motives through work [47], the “meaning of work” should be explored first. This study can be said to be highly meaningful in that it represents the first attempt to study the subjectivity of “the meaning of work” with NGO employees.

This study examined how Generation MZ members working for Korean NGOs perceive the meaning of work, what role work plays in individuals’ lives, and what reinforces the meaning of work for them. The limitations and suggestions of this study are as follows: First, since most of the study subjects worked in the field of fundraising marketing, there may be limitations in generalising the results of this study to all Generation MZ employees of NGOs. Thus, additional studies covering various occupational groups are necessary. Second, to increase organisational satisfaction by type and create appropriate corporate cultures, follow-up studies on context and motivational variables involving in-depth interviews and additional studies with individuals by type are needed. Third, to increase our understanding of Generation MZ employees working for NGOs, Generation MZ’s general perceptions of the “meaning of work” must be compared and studied to identify the differences. The working environments of NGOs should differ by country and culture, as should members’ perceptions of the meaning of work. It is hoped that various quantitative and qualitative follow-up studies will continue to provide meaningful implications for human resource development and the strengthening of organisational power suitable for the organisational environment of NGOs that work for the public interest of our society. 

## Figures and Tables

**Table 1:** Definitions of the meaning of work.

Dimension	Author	Meaning of Work
Cognitive dimension	[24]	Not only the reward for the work carried out by the person but also the alignment of the purpose, values, and relationships pursued in life
	[25]	Cognitive evaluation subjectively given by the person to the work being done by the individual
	[18]	What work means in individuals’ lives and what roles it occupies in their lives
Cognitive-behavioral dimension	[26]	Regarding one’s work as an important and subjective experience of finding oneself and growing through work while the work is positively influencing others or society
	[27,28]	The totality of the beliefs, values, motives, importance, and purpose that an individual has about work—that is, a comprehensive attitude of cognition, emotion, and behaviour toward work
	[29]	Meaningful work not only brings about economic rewards but also makes individuals’ lives meaningful

**Table 2:** Study process.

**Stage**		**Study Process**
**Stage 1**	Construction of Q populations	- Literature review, including review of newspaper articles and various reports (95) - Written and in-depth interviews with three Generation MZ employees of NGOs (37) - Construction of a total of 113 Q populations
**Stage 2**	Q sample selection	- Extraction of Q samples by the principal researcher using the non-structural method - Review by two fellow doctoral students taking the Q Methodology class - Final review by two Q methodology experts - Selection of a total of 40 Q samples
**Stage 3**	P sample selection	- 24 Generation MZ employees working for NGOs
**Stage 4**	Q sorting	- Forced distribution method by P samples - 11-point scale
**Stage 5**	Data processing and analysis	- KADE v2.0.0 - Application of principal components and varimax rotation

**Table 3:** Q statements.

No.	Category	Statement
**Q1**	Economics	Work is a means of livelihood.
**Q2**	Economics	Work is a means of preparation for old age.
**Q3**	Economics	Work enables economic independence.
**Q4**	Economics	I want to make money to live a life in which I can retire as soon as possible.
**Q5**	Economics	The higher a job’s salary, the better.
**Q6**	Economics	I like stable jobs.
**Q7**	Economics	If I were born with a silver spoon in my mouth, I would not bother to work.
**Q8**	Quality of life	You only have to work as much as you get paid.
**Q9**	Self-realisation	I am happy only when my work fits my values.
**Q10**	Self-realisation	Work enables me to feel that I am a person of value.
**Q11**	Self-realisation	It is hard for me to feel that I am growing through my work.
**Q12**	Self-realisation	My work helps me understand myself better.
**Q13**	Self-realisation	Work is an opportunity to feel a sense of achievement.
**Q14**	Self-realisation	Work is a place to express one’s aptitudes and interests.
**Q15**	Self-realisation	Work is a process to build a desired career.
**Q16**	Self-realisation	Work is an opportunity to try new things.
**Q17**	Social	Work gives me a feeling of satisfaction that I am contributing to society and people.
**Q18**	Self-realisation	Work is like studying while being paid.
**Q19**	Happiness	Work should be meaningful.
**Q20**	Happiness	Work is a tonic for life.
**Q21**	Other	I like the kind of jobs that I can do until retirement without worrying about losing the job or being fired.
**Q22**	Happiness	Work is a source of stress.
**Q23**	Happiness	Work cannot make me remain the way I am.
**Q24**	Happiness	Work should be interesting.
**Q25**	Social	I am recognised for the work I do.
**Q26**	Social	My value is not evaluated (defined) by the outcome of my work.
**Q27**	Economics	If I could keep receiving unemployment benefits, I would not bother trying to work.
**Q28**	Social	Work is a place to meet, communicate and exchange with various people.
**Q29**	Social	It is important to work with people who share your values and beliefs.
**Q30**	Social	One’s job determines one’s social status.
**Q31**	Happiness	I am happiest when I work with a sense of duty.
**Q32**	Social	I want to do work for which I am respected by people.
**Q33**	Happiness	Work enhances my self-esteem.
**Q34**	Quality of life	Work is only a part of life.
**Q35**	Quality of life	To work well, I need the time to be fully invested in myself.
**Q36**	Quality of life	I work only enough to maintain a work–life balance.
**Q37**	Other	I want to continue working beyond retirement age.
**Q38**	Other	It’s important that I do work that fits my ability.
**Q39**	Social	To be successful, you should work hard.
**Q40**	Other	My daily life is my top priority.

**Table 4:** Demographic characteristics of P samples.

Division	Categories	N (Total 24)
Gender	Male	4
	Female	20
Age	20s	5
	30s	13
	40s	6
Number of years of continuous service	1–3 years	8
	4–7 years	9
	8–10 years	6
	10 years or more	1
Professional field	Marketing (Brand/Fundraising/Sponsor)	18
	Business (Overseas/Domestic)	3
	Management support	3

**Table 5:** Eigenvalues and explained variances by type.

Content	Type 1	Type 2	Type 3	Type 4
Eigenvalue	9.8081	2.5302	1.5321	1.389
% Explained variance	0.41	0.11	0.6	0.6
Cumulative % explained variance	0.41	0.52	0.58	0.64

**Table 6:** Correlation coefficients between individual types.

Type	Type 1	Type 2	Type 3	Type 4
Type 1	1			
Type 2	0.583	1		
Type 3	0.5348	0.5906	1	
Type 4	0.4911	0.3997	0.4603	1

**Table 7:** Subjects and factor weights by type.

Type	No.	Gender	Age	Professional Field	Factor Weight
Type 1	P15	Female	30s	Marketing	10
(*n* = 4)	P24	Female	20s	Business	9.9729
	P18	Female	30s	Marketing	9.5839
	P03	Female	30s	Marketing	7.8218
Type 2	P20	Female	20s	Marketing	12.9776
(*n* = 7)	P19	Male	30s	Marketing	11.8750
	P16	Female	40s	Management support	10.5480
	P05	Female	30s	Marketing	9.9147
	P04	Female	30s	Marketing	−8.4707
	P11	Female	40s	Business	8.2646
	P06	Female	40s	Marketing	8.1669
Type 3	P10	Male	30s	Marketing	17.5581
(*n* = 3)	P22	Female	30s	Marketing	12.1431
	P02	Female	30s	Management support	11.8802
Type 4	P13	Female	30s	Marketing	20.3488
(*n* = 7)	P12	Female	40s	Marketing	11.9975
	P07	Female	30s	Marketing	11.6653
	P23	Female	20s	Business	8.6810
	P09	Male	40s	Marketing	8.5235
	P21	Female	30s	Marketing	7.1098
	P01	Female	40s	Marketing	6.4764

**Table 8:** Standard scores by Type 1 item.

No.	Statement	Z-Score
26	My value is not evaluated (defined) by the outcome of my work.	1.759
9	I am happy only when my work fits my values.	1.655
16	Work is an opportunity to try new things.	1.617
3	Work enables economic independence.	1.320
35	To work well, I need the time to be fully invested in myself.	1.288
24	Work should be interesting.	1.102
31	I am happiest when I work with a sense of duty.	1.020
37	I want to continue working beyond retirement age.	1.003
23	Work cannot make me remain the way I am.	−1.035
40	My daily life is my top priority.	−1.063
11	It is hard for me to feel that I am growing through my work.	−1.155
22	Work is a source of stress.	−1.250
4	I want to make money to live a life in which I can retire as soon as possible.	−1.289
30	One’s job determines one’s social status.	−1.429
7	If I were born with a silver spoon in my mouth, I would not bother to work.	−1.742
27	If I could keep receiving unemployment benefits, I would not bother trying to work.	−1.853

**Table 9:** Standard scores by Type 2 item.

No.	Statement	Z-Score
9	I am happy only when my work fits my values.	1.814
10	Work enables me to feel that I am a person of value.	1.777
17	Work gives me the feeling of satisfaction that I am contributing to society and people.	1.679
13	Work is an opportunity to feel a sense of achievement.	1.410
12	My work helps me understand myself better.	1.362
14	Work is a place to express one’s aptitudes and interests.	1.269
38	It’s important that I do work that fits my ability.	−1.259
11	It is hard for me to feel that I am growing through my work.	−1.316
8	You only have to work as much as you get paid.	−1.438
4	I want to make money to live a life in which I can retire as soon as possible.	−1.449
23	Work cannot make me remain the way I am.	−1.549
27	If I could keep receiving unemployment benefits, I would not bother trying to work.	−1.566
7	If I were born with a silver spoon in my mouth, I would not bother to work.	−2.016

**Table 10:** Standard scores by Type 3 item.

No.	Statement	Z-Score
9	I am happy only when my work fits my values.	2.028
29	It is important to work with people who share your values and beliefs.	1.846
24	Work should be interesting.	1.543
31	I am happiest when I work with a sense of duty.	1.541
19	Work should be meaningful.	1.485
28	Work is a place to meet, communicate, and exchange with various people.	1.284
38	It’s important that I do work that fits my ability.	1.225
8	You only have to work as much as you get paid.	−1.118
27	If I could keep receiving unemployment benefits, I would not bother trying to work.	−1.163
21	I like the kind of jobs that I can do until retirement without worrying about losing the job or being fired.	−1.172
18	Work is like studying while being paid.	−1.177
7	If I were born with a silver spoon in my mouth, I would not bother to work.	−1.231
2	Work is a means of preparing for old age.	−1.233
11	It is hard for me to feel that I am growing through my work.	−1.543
23	Work cannot make me remain the way I am.	−1.907

**Table 11:** Standard scores by Type 4 item.

No.	Statement	Z-Score
34	Work is only a part of life.	2.201
9	I am happy only when my work fits my values.	1.820
29	It is important to work with people who share your values and beliefs.	1.753
35	To work well, I need to be fully invested in myself.	1.370
3	Work enables economic independence.	1.338
15	Work is a process to build a desired career.	1.197
28	Work is a place to meet, communicate, and exchange with various people.	1.033
31	I am happiest when I work with a sense of duty.	−1.152
37	I want to continue working beyond retirement age.	−1.404
21	I like the kind of jobs that I can do until retirement without worrying about losing the job or being fired.	−1.405
7	If I were born with a silver spoon in my mouth, I would not bother to work.	−1.629
18	Work is like studying while being paid.	−1.658
27	If I could keep receiving unemployment benefits, I would not bother trying to work.	−1.970

**Table 12:** Consensus statements.

No.	Statement	Type 1 Z-Score	Type 2 Z-Score	Type 3 Z-Score	Type 4 Z-Score
9	I am happy only when my work fits my values.	1.655	1.814	2.028	1.82
3	Work enables economic independence.	1.32	0.98	0.56	1.34
7	If I were born with a silver spoon in my mouth, I would not bother to work.	−1.742	−2.02	−1.23	−1.629
11	It is hard for me to feel that I am growing through my work.	−1.155	−1.32	−1.54	−0.79
27	If I could keep receiving unemployment benefits, I would not bother trying to work.	−1.853	−1.566	−1.16	−1.97

**Table 13:** Meaning–dimension relationships of work desired by individual types and individuals.

Division	Job Orientation	Career Orientation	Calling Orientation
Type 1: Work is my opportunity to grow	O	O	O
Type 2: Work enables me to realise my value			O
Type 3: Work is an interesting experience			O
Type 4: Work is just a part of life	O	O	

## Data Availability

Data from the study are available upon request.
